# Growth and energy and protein intake of preterm newborns in the first year of gestation-corrected age

**DOI:** 10.1590/S1516-31802003000100002

**Published:** 2003-01-02

**Authors:** Júlia Laura Delbue Bernardi, Ana Lúcia Goulart, Olga Maria Silverio Amancio

**Keywords:** Premature infants, Growth, Nutritional evaluation, Diet, Prematuros, Crescimento, Avaliação, Nutricional, Dieta

## Abstract

**CONTEXT:**

There are few longitudinal studies that analyze the growth and nutritional status parameters of children born prematurely.

**OBJECTIVE:**

To evaluate the growth and dietary in-take of preterm newborns in the first year of gestation-corrected age.

**DESIGN:**

Prospective clinical study.

**SETTING:**

Tertiary care hospital.

**PATIENTS:**

19 children (7 male) who were born prematurely, with birth weight between 1000g and 2000g, which was adequate for the gestational age.

**PROCEDURES:**

At 3, 6, 9 and 12 months of gestation-corrected age, children were evaluated in relation to weight, height and cephalic perimeter, using the National Center for Health Statistics as the standard reference, and the Rozalez-Lopez and Frisancho standards for brachial perimeter and triceps and subscapular skinfolds. The calculated dietary intake was compared to the Recommended Dietary Allowances.

**MAIN MEASUREMENTS:**

The Z score was calculated for the weight/age, height/age and weight/height relationships, and the percentiles of the perimeters and skinfolds were considered. Dietary intake records were made using the 24-hour Dietary Recall and the Food Frequency Intake Questionnaire methods. The Virtual Nutri software was used to calculate energy and protein intake.

**RESULTS:**

The weight/age, height/age and weight/height relationships and the brachial perimeter and triceps skinfold were statistically greater in the first semester in relation to the second. The cephalic perimeter remained above the 50 percentile for the ages studied and there was no difference in the subscapular skinfold between the first and second semesters, remaining below the 50 percentile. The calorie and protein intake, although statistically lower in the first than in the second semester, always remained above the recommended.

**CONCLUSIONS:**

The pace of growth is greater in the first semester than in the second, not reaching the standard expected for full-term newborns, with the exception of the cephalic perimeter, which remains adequate. Calorie/protein intake shows an inverse relationship with growth speed, remaining above the recommended for full-term newborns, although with difficulty in depositing subcutaneous fat, in spite of the high caloric intake.

## RESUMO

**CONTEXTO:**

Atualmente há estudos longitudinais limitados que definem parâmetros de crescimento e estado nutricional de crianças nascidas prematuras.

**OBJETIVO:**

Avaliar o crescimento e a ingestão dietética em recém-nascidos pré-termo no primeiro ano de idade corrigida.

**TIPO DE ESTUDO:**

Estudo clínico prospectivo.

**LOCAL:**

Hospital de cuidados terciários.

**PACIENTES:**

19 crianças (sete do sexo masculino) nascidas prematuras, adequadas para a idade gestacional, com peso de nascimento entre 1.000 g e 2.000 g, acompanhadas aos 3, 6, 9 e 12 meses de idade corrigida.

**PROCEDIMENTOS:**

Aos 3, 6, 9 e 12 meses de idade corrigida, as crianças foram avaliadas quanto ao peso, estatura e perímetros utilizando-se, como padrão de referência, o National Center of Health Statistics e quanto à circunferência braquial e às dobras cutâneas triciptal e subescapular, utilizandose o padrão de Ronalez-Lopez e de Frisancho. A ingestão dietética calculada foi comparada às Recommended Dietary Allowances.

**RESULTADOS:**

As relações peso/idade, estatura/idade, peso/estatura, circunferência braquial e dobra cutânea do tríceps foram estatisticamente maiores no primeiro semestre em relação ao segundo. O perímetro cefálico manteve-se acima do percentil 50 nas idades estudadas e a dobra cutânea subescapular não mostrou diferença entre o primeiro e o segundo semestres, mantendo-se abaixo do percentil 50. A ingestão de calorias e proteínas, apesar de estatisticamente menor no primeiro do que no segundo semestre, permaneceu sempre maior do que o recomendado.

**CONCLUSÕES:**

O ritmo de crescimento mostra maior velocidade no primeiro semestre do que no segundo, não atingindo o padrão esperado para os recém-nascidos a termo, com exceção do perímetro cefálico, que se mantém adequado. A ingestão de calorias/proteínas mostra relação inversa com o ritmo de crescimento, permanecendo acima do recomendado para nascidos a termo, havendo, porém, dificuldade de deposição de gordura subcutânea, apesar da alta ingestão calórica.

## INTRODUCTION

Although both morbidity and mortality among children born prematurely are still high, especially in developing countries, progress in their obstetric and neonatal care has been contributing towards ensuring preventive strategies and survival therapeutics.

In Brazil, in 1999, 8.5% of the children born in the state of São Paulo had a birth weight of less than 2500 g, and 6.54% were born before 37 weeks of gestational age.^1^ American statistics^2^ show an incidence of 7.5% of low birth weight and 1.4% for very low birth weight, over the whole country.

The predisposing factors for prematurity are related to maternal and obstetrics problems and fetal conditions.^3,4^ In this way, prenatal examinations are a preventive tool for this condition, in association with improvements in socioeconomic level and the mother's education level.

Problems resulting from prematurity,^5^ such as anemia, chronic pulmonary disease and neurological and developmental disorders can interfere with growth, which is an important marker of health. In relation to intrauterine growth, the third three-month period of gestation seems to be the most critical for fetal weight gain, and therefore, children born before full-term lose part of this gain. In addition to this, they go through an initial weight loss at birth, which on average reaches 15%,^6^ and is greater and more prolonged with lower gestational ages and the presence of neonatal intercurrences. In such a situation, future growth may be compromised.

Premature newborns constitute a group at high risk of delays and deviations in growth. This has been stimulating a need for knowledge of their growth pattern, which today is not well established in the literature. There is controversy among existing studies, without definition of whether growth acceleration occurs, or whether these children will reach the standard expected for full-term newborns of the same age.^7,8^

To evaluate the process of postnatal somatic growth, anthropometric data that will reflect the reality of this pattern, such as weight, height, perimeters and skinfold curves, should be used. In Brazil, there are not many longitudinal studies on the growth of preterm newborns. The study by Goulart et al.(1996),^9^ analyzing 61 premature newborns, concluded that those children seemed not to reach the expected growth for full-term newborns by the end of the first year, even when considering the gestation-corrected age, to allow for the degree of prematurity. In the international literature, Babson's study (1970)^10^ is a classic, which reported increased speed of growth for the cephalic perimeter, while the curves remained parallel in relation to weight and height, but below of those of children born at full term.

In the 20-week-old human fetus, the anatomical differentiation of the digestive system is considered significant, but functional maturity remains limited up to the 26^th^ week of gestation.^11^ Because of this immaturity, there have been reports that there may be complications in the nutritional handling of such children, especially in the first months of life, since their needs are based on the intrauterine accumulation rate,^12,13^ which is high. Nonetheless, what has been done is to use the Recommended Dietary Allowances^14^ developed for full-term newborns, in view of the fact that, in the literature, no specific recommendations for premature newborns are to be found.

In this way, it can be seen that such children, whose growth dynamics seem to be differentiated in their first year of life, should have closer nutritional follow-up, since the consequences of the nutritional decisions taken during this period can last for their whole lives.

Consequently, the aim of this study was to evaluate the growth and dietary intake of preterm newborns in the first year of gestation-corrected age.

## METHODS

This was a prospective clinical study on 19 preterm children, 7 of whom were male, born at the Hospital São Paulo and followed up at the premature infants outpatient service of the same hospital, which is a public institution forming part of the Universidade Federal de São Paulo, between March 1998 and September 1999.

The gestational age was calculated via the Naegelle method^15^ and confirmed through the newborn clinical evaluation using the Dubowitz,^16^ Capurro^17^ or Ballard^18^ methods.

The premature newborns selected for the study had a gestational age of less than 37 weeks,^19^ adequate development for their gestational age, and did not present any severe disease (genetic syndromes, level III or IV perior intraventricular hemorrhages, or chronic pulmonary disease) that could interfere with the growth process.

These premature newborns were followed up during their first year of life, considering the gestation-corrected age to allow for the degree of prematurity^10^ (the postnatal age after subtracting the number of weeks lacking for forty weeks of intra-uterine life to be completed). This allows a more adequate comparison to be made with full-term newborns.

On the 4 occasions that the newborns were attended (at 3, 6, 9 and 12 months of gestation-corrected age), an anthropometric evaluation was made, the dietary intake record was obtained and nutritional guidance was given. For the anthropometric measurements, the recommended techniques were used.^20^ The weight, height and cephalic perimeter were compared with the National Center of Health Statistics (NCHS) values.^21^ The weight-forage (W/A), height-for-age (H/A) and weight-for-height (W/H) relationships were considered, with Z scores calculated via the Epi-Info version 6.0 software.^22^ The triceps and subscapular skinfolds and the brachial perimeter were compared with the Rosales-Lopez^23^ and Frisancho^24^ standards.

The dietary intake record was obtained via the 24-hour dietary recall^25^ and the food frequency intake Questionnaire methods.^26^ Energy and protein intakes were calculated using the validated and standardized Virtual Nutri^27^ software. The calculated dietary intake was compared with the Recommended Dietary Allowances (RDA).^14^

Nutritional guidance^28^ for the age ranges of 3 to 6, 6 to 9 and 9 to 12 months was explained orally and handed out in written form to the mothers.

Prior written consent was obtained from the parents and the study was approved by the Committee for Ethics in Research of the Universidade Federal de São Paulo/Hospital São Paulo.

*Statistical Analysis.* Friedman's variance analysis^29^ by ranks was used, complemented with Dunn's Multiple Comparison Test,^30^ adopting alpha £ 5%.

## RESULTS

In the Table it can be seen that the three relationships, weight-for-age, height-for-age and weight-for-height, showed statistical differences when analyzed over the course of the first year of gestation-corrected age. Thus, the median Z score of the weight-for-age relationship was statistically greatest at the 3^rd^ month in relation to the others (p < 0.0001).

**Table:** Median Z -scores for the anthropometric relationships, the percentiles for the perimeters and skinfolds, and the daily calorie (kcal) and protein (g) intakes by premature newborns at 3, 6, 9 and 12 months of gestation-corrected age

Gestation-corrected age (months)					p values
	3 n = 19	6 n = 19	9 n = 19	12 n = 19	
W/A	-0.34^a^ (-1.7/1.8)	-0.81^b^ (-2.6/1.8)	-0.95^b^ (-3.0/1.7)	-0.80^b^ (-3.0/1.3)	<0.0001
H/A	0.28^a^ (-2.7/1.8)	-1.24^b^ (-2.2/0.6)	-0.91^b^ (-2.8/1.2)	-0.60 (-3.3/1.28)	<0.002
W/H	0.14^a^ (-1.0/2.3)	0.16^a^ (-1.9/1.9)	0.22 (-2.2/1.2)	0.9^b^ (-1.7/0.9)	<0.0001
Cephalic perimeter	75^a^ (15-97.5)	75^a^ (5-97)	50^b^ (3-75)	60^b^ (3-97.5)	<0.001
Brachial perimeter	75^a^ (50-75)	50 (10-97)	60^a^ (3-97,5)	30^b^ (5-90)	<0.001
Triceps skinfold	25^a^ (3-90)	30^a^ (2.5-97.5)	15 (2.5-97.5)	10^b^ (2.5-75)	<0.023
Subscapular skinfold	15 (2.5-97.5)	10 (2.5-75)	25 (2.5-90)	15 (2.5-75)	0.866
Calories^1^ (KCal)	835^a^ (450-1.917) | * |	1.051^a^ (558-2.041) | * |	1.561^b^ (707-2.947) | * |	1.657^b^ (836-2.974) | * |	<0.0001
RDA^2^ (KCal)	570	712.5	890	1005	
Proteins^1^ (g)	22^a^ (7-91)	40^a^ (9-57.5)	53,5^b^ (34-98)	63^b^ (45-100.5)	<0.0001
	| * |	| * |	| * |	| * |	
*RDA^2^*g – proteins	13	14	14	16	

*W/A = weight-for-age; H/A = height-for-age; W/H = weight-for-height; () variation in the values found; p: descriptive level of Dunn's multiple comparison test; 1. 24-hour dietary recall and food frequency intake questionnaire methods; 2. Recommended Dietary Allowances; Line with different superscribed letters: with statistical difference*

The median Z score of the height-for-age relationship showed a statistical difference at the 3^rd^ month, in relation to the 6^th^ and 9^th^ months (p < 0.002), and, for the weight-for-height relationship it was significantly greater at the 3^rd^ and 6^th^ months, than at the 12^th^ month (p < 0.0001).

The median percentiles of the cephalic and brachial perimeters showed that the first parameter was statistically greater in the first semester, at the 3^rd^ and 6^th^ months, than in the second semester, at the 9^th^ and 12^th^ months (p < 0.001), while the brachial perimeter was significantly greater at the 3^rd^ and 9^th^ months than at the 12^th^ month (p < 0.001).

With regard to the skinfolds, the median percentile for the triceps was greater at the 3^rd^ and 6^th^ months than at the 12^th^ month (p < 0.023), while for the subscapular, it did not show any significant difference between the studied months (p = 0.866).

The median daily calorie and protein in-takes showed values that were statistically greater in the second semester in relation to the first, both for calories (p < 0.0001) and proteins (p < 0.0001), with no significant difference between the 3^rd^ and 6^th^ months and between the 9^th^ and 12^th^ months. The comparison of the median intake with the recommendation (RDA) showed that it was statistically greater than the recommended, both for calories and proteins, on the four occasions studied, and always with p < 0.0001.

## DISCUSSION

Children of low birth weight experience different growth patterns in relation to children of normal weight at birth, and can more easily become "thin and short".^31^ In this respect, it has been suggested that children born under this condition suffer postnatal growth deficit in the first year of life.^32^ In our study, 26.3% of the children presented a weight deficit at 12 months, while 21% presented severe height deficit (Table 1). This therefore concords with reports in the literature that the recovery in height occurs earlier than body weight recovery.

The growth evolution of preterm newborns in relation to full-term newborns is a question that has presented contradictory results in the literature. Some studies have reported that the pattern for premature newborns is inferior,^33,34^ while others have brought out evidence of similar growth patterns for the two groups.^35,36^ The differences found could be due to the methodologies in relation to the characteristics of the premature infants, the study duration, or the socioeconomic level, among other reasons.

With regard to the cephalic perimeter, this is the first growth parameter to show acceleration, and is followed by the stature and weight. The evolution of the cephalic perimeter is greatly increased in the beginning, and is discordant with the other variables, especially during the first 3 months. This is the parameter that is closest to the standard expected for full-term newborns.^37^ This greater speed of growth is confirmed in this study, since this was the anthropometric variable that presented the smallest percentile decrease in the second semester of gestation-corrected age, and it was maintained within the standards considered normal for full-term newborns.

Furthermore, for the methods that represent fat and muscle deposits (skinfolds and perimeter, respectively),^10,15^ we found change in percentiles towards lower ranges with increasing age. However, at 12 months of gestation-corrected age, the median percentiles for the brachial perimeter, triceps skinfolds and subscapular skinfold showed that there was an improvement in muscle mass and less fat deposition. Similar results have been reported, and two hypotheses have been suggested in order to explain this type of result. The first is that the lack of fat deposition may be due to the high energy cost for the accretion of this nutrient. The second is that these children may not be able to ingest caloric quantities beyond their basal needs for maintenance, which is an in-take insufficient to allow fat deposition. For this, their maintenance needs would be increased or there would be excessive caloric loss in feces.^38^

The calorie and protein intakes showed an inverse relationship with the speed of growth, being smaller in the beginning (3 and 6 months) and increasing greatly over time (9 and 12 months), always in significantly greater quantities than the recommended for full-term newborns. Similar results were described in 1991.^39^ These results may be a consequence of the following facts. Premature newborns present more accentuated growth in the first months of life in relation to later months.^40^ They also have immaturity of the organ systems with consequent compromising of the absorption mechanisms, in such a way that it is not possible for all the ingested energy to be metabolized.^41^ Finally, they show difficulty in depositing nutrients, which is confirmed by the inverse relationship between energy and protein ingested, and the energy stored during growth, which has the capability for causing a decrease in the speed of growth.^42^

Another consideration concerning nutrient intake is in relation to the fact that there is a lack of nutritional recommendations for premature newborns in the first year of life. The energy expenditure of these children may be not the same as for children born full-term, since their energy stores are smaller at birth and therefore their needs and the consequent recommendations should be specific.

However, the hypothesis that the calorie and protein intake results in our study could have been a consequence of the type of dietary evaluation conducted cannot be discarded. The 24-hour dietary recall could have been overestimated by the mother or person responsible and, for this reason, it would have been interesting for the dietary intake analysis to have been conducted either at home or for a longer period, for instance by means of a consecutive 4-day dietary record for each consultation.

In this way, further research is necessary for a better understanding of the growth and nutritional status of premature infants, preferably with more frequent evaluations and dietary guidance, including at a domestic care level Studies should also consider the minerals and vitamins related to growth and the pertinent biochemical evaluation.

## CONCLUSIONS

From the results obtained from the evaluation of the prematurely born children, with follow-up in the first year of gestation-corrected age, it can be concluded that the pace of growth shows greater speed in the first semester than in the second. Their growth does not reach the standard expected for full-term newborns, with the exception of the cephalic perimeter, which is adequately maintained. Calorie and protein intake shows an inverse relationship with the pace of growth, while remaining above the recommended for full-term newborns. There is, however, difficulty in depositing subcutaneous fat, in spite of the high caloric intake.
